# Intensive care decision‐making, survival and dying well: a mixed methods study with people who have been intensive care patients

**DOI:** 10.1111/anae.70285

**Published:** 2026-07-09

**Authors:** Thomas Donaldson, Kirsty Keywood, Lucy Frith, Søren Holm

**Affiliations:** ^1^ Centre for Social Ethics and Policy, School of Social Sciences, University of Manchester Manchester UK

**Keywords:** decision‐making, dying, intensive care, patient experience, survival

## Abstract

**Introduction:**

Admission to the ICU involves experiential burdens and a risk of a negative dying experience if patients do not survive their critical illness. Many patients who are critically unwell, or their surrogate decision‐makers, must decide about accepting ICU treatment without knowing what it is like. People who have been through ICU treatment can provide useful insights into what it might be like to die in ICU and what chance of survival makes ICU treatment acceptable to them.

**Methods:**

This study utilised a mixed methods approach involving questionnaires and semi‐structured interviews which were analysed using a reflexive thematic analysis approach. This was done to provide a deep and rich analysis of how participants' experiences of ICU treatment and end‐of‐life wishes affected their reflections about what it might be like to die on an ICU, and their willingness to accept ICU treatment again in the future.

**Results:**

Twenty‐six people who had experienced ICU as patients were interviewed. Whilst some positive experiences were reported, participants thought the negative experiences associated with ICU made it likely that dying on ICU would be a negative dying experience. Being a patient in an ICU involved a confrontation with mortality, loss of control and total dependence, and was described as a transformative experience. Participants considered the chance of survival to be an important consideration in shared decision‐making with ICU clinicians about ICU treatments. The median chance of survival that made ICU treatment acceptable was 30%.

**Discussion:**

An understanding of the experiences associated with being a patient in the ICU can inform how patients and clinicians consider the risk–benefit analysis of ICU treatment. The experiential harms of ICU admission and the risk of ICU treatment resulting in a negative dying experience may outweigh the survival benefit that ICU treatment will offer a patient when there is a low chance of survival.

## Plain Language Summary

**What we did:**

We spoke to 26 people who had been treated in an intensive care unit (ICU). They completed questionnaires and took part in interviews about their experiences. We asked them what it was like to be in ICU, how they felt about the possibility of dying there, and whether they would choose ICU treatment again in the future.

**Why did we do it:**

People who are very ill or their families, often have to decide whether to accept ICU treatment without knowing what it is really like. We wanted to learn from people who had experienced ICU care so that future patients and doctors can make more informed decisions together.

**What we found:**

Many people described ICU as a life‐changing experience. Although some had positive memories, many also remembered difficult experiences, including feeling frightened, losing control and depending completely on others for care. Most participants felt that dying in ICU would probably be a difficult experience. When thinking about whether they would choose ICU treatment again, the chance of survival was very important. On average, people felt that ICU treatment was worthwhile if they had at least about a 30% chance of surviving. These findings suggest that the benefits of ICU treatment need to be carefully balanced against the burdens of treatment, especially when the chance of survival is low.

## Introduction

There is growing demand for treatment in the ICU for patients with low chances of surviving [1, 2]. Alongside this, there is an increasing proportion of older patients on ICUs [2, 3]. Comorbidity is common in patients who are older, and poor outcomes from ICU treatment have been linked to comorbid conditions [4, 5]. This could result in more patients dying on ICUs who, by definition, cannot subsequently report their experiences [6]. Patients who are sedated on ICUs often appear unconscious; however, many people who have been patients on ICUs recall negative experiences including pain; disturbing dreams; procedures; thirst; noise; invasive devices; panic; fear; helplessness; lack of control; and thinking they were dying [7, 8, 9]. These experiences of what it is like to receive ICU treatment are likely to be shared by all patients on ICUs, both those who survive their critical illness and those who do not [6]. Consequently, dying whilst receiving ICU treatment is likely to be a very distressing experience [10]. For most people, hospital is not the preferred place of death [11].

There is significant clinical uncertainty in predicting which patients will survive a critical illness [12]. However, the potential harms inherent in ICU treatment can easily be overlooked by patients, families and clinicians when time‐critical decisions must be made regarding clinical management. Studies have explored both whether people who have been patients on ICUs retrospectively agreed with the decision to provide them with ICU treatment, as well as whether they would have such treatment again [13, 14]. However, more research is needed on the perspectives of patients relating to ICU decision‐making in the context of clinical uncertainty. This topic is relevant to the ICU research priorities as identified by the James Lind Alliance [15], specifically ‘*how can we predict who will benefit from intensive care before admission and during treatment in the ICU?*’

It is not possible to ask patients who did not survive their critical illness about their dying experience or whether they retrospectively agreed with the decision to provide ICU treatment. However, asking people who have previously been patients on ICUs about what chance of survival would make ICU treatment acceptable to them could provide an important guide for ethical decision making. Many patients who are critically unwell faced with the decision of whether to accept ICU treatment have no previous experiences of what it is like. The experiences of people who have been patients on ICUs are, therefore, a valuable source on information for those facing this important life or death decision. This information could be used to guide ICU decision‐making and to better inform patients about the chance of survival that makes the suffering involved in ICU treatment acceptable to patients.

A key question in the provision of ICU treatment is: “*at what chance of survival is this treatment ethically justifiable?*” An adequate answer to this question will require consideration of both the quantitative benefits of the treatment, such as survival, and the qualitative harms, including suffering and the risk of exposure to a negative dying experience [16]. In order to help inform ICU decision‐makers and people considering ICU treatment who have not experienced it [10], this study aimed to investigate the factors affecting the willingness of people who have previously experienced ICU treatment to accept ICU treatment again and the chance of survival that makes the suffering involved in ICU treatment acceptable to them.

## Methods

Research Ethics Committee opinion was obtained from the North West – Liverpool Central Research Ethics Committee. The Critical Care Support Network provided patient and public input into the development of the study methodology, interview guide and research protocol.

This study utilised a mixed‐methods design [17], combining qualitative and quantitative methods, based on a ‘pragmatic approach’ [18]. This offered a suitable methodological approach to answering the research question and allowed for triangulation between thematic data from semi‐structured interviews and quantitative data from questionnaires. The quantitative methodology provided a rich complementary data source to the qualitative findings, supporting a ‘thick description’ of the context of the study to facilitate interpretation and enhance analytic generalisations [19].

The recruitment strategy aimed to generate a purposeful sample of informants who had undergone the experience of being a patient on an ICU as they represented a source of information rich data [20]. To achieve this, recruitment occurred through the provision of participant information sheets to adults who had been patients on ICU, both in ICU review clinics (by the individual's clinical team) and through ICU support groups (Critical Care Survivors Network and ICU Steps). No compensation was made available for participating in this study.

We discussed the study and risks of participation with potential participants before seeking informed consent, including for audio recording and anonymised dissemination of results. If they wished to participate, written consent was gained. As part of the anonymisation protocol each participant was assigned a random identification number between 1 and 50.

Participants undertook one‐on‐one, semi‐structured, in‐depth interviews [21]. Emotional burden on participants is a known hazard of qualitative research in healthcare [22]. Individual interviews provided a more supportive context than focus groups and participants received debrief sheets with sources of support. We conducted interviews either face‐to‐face or via videoconferencing (Zoom Workplace, Zoom Communications Inc., San Jose, CA, USA) as requested by participants. The interviews were conducted by the principal investigator (TD) following a standardised interview structure to ensure consistency.

Interview audio recordings were transcribed by a University of Manchester approved transcribing service. Transcripts were manually checked against the audio recordings for accuracy and to remove identifiable information. Thematic analysis was undertaken using NVivo (version 14, Lumivero, Denver, CO, USA) qualitative data analysis software by TD under the supervision and monitoring of his research supervisor SH, who performed a check of the analysis by independently coding a sample of the transcripts. Codes generated from the data were discussed within the research team and organised into unifying themes. Transcripts were re‐reviewed to ensure comprehensive analysis. Data collection was stopped when thematic saturation was identified, when three consecutive interviews generated no new themes [20].

The questionnaires included assessment of the ICU experiences of participants through the ICU Memory Tool [23]. The functional status of participants before and after ICU admission was measured through the EuroQol 5 Dimension 5 Level (EQ‐5D‐5L) tool [24], which assesses five domains of quality‐of‐life: mobility; self‐care; usual activities; pain/discomfort; and depression/anxiety. Preferences of participants for end‐of‐life care were assessed using the Concept of a Good Death Measure [25], which measures factors that people consider important to a good death. Participants were also asked to assess ICU as a dying experience using the Quality of Dying and Death questionnaire, which is used to evaluate end‐of‐life experiences [26].

Further integration of quantitative and qualitative factors was undertaken by asking “*what chance of survival would make you willing to go through that experience again?*” This required participants to use their qualitative experiences to guide their choice of chance of survival at which they would accept ICU treatment (which is a quantifiable decision).

In order to account for the influence of the perspectives and values of interviewers and participants in the interview process, reflexive thematic analysis was chosen to identify patterns of meaning and themes across the dataset [27]. Accounting for subjectivity the of interviewer and participant mirrored the importance of accounting for the values of both in ICU decision‐making. The principal investigator (TD) is a consultant anaesthetist, with experience of working on ICUs, although not in his current practice. He reflected upon these experiences in a study diary throughout preparing and carrying out the study to acknowledge potential impact on the data, provide clarity regarding how data were interpreted, and ensure certain knowledge was not privileged [28].

## Results

Twenty‐seven participants consented to participate in the study and 26 completed interviews between October 2023 and October 2025. One participant withdrew from the study and did not complete the interview. Thematic saturation was identified after analysis of 26 transcripts. The median interview duration was 57 min. Twenty‐five participants completed the ICU Memory Tool, EQ‐5D‐5L and Good Death Measure and 21 completed the Quality of Dying and Death questionnaire.

There were 15 male participants and 11 female participants; 25 identified as White and one identified as Black. The distribution of ages is reported in Table 1. Twenty‐four of the participants provided information about the cause of their admission and their length of stay on ICU: 21 admissions to ICU were emergency medical admissions and three were emergency surgical admissions. Three participants had a duration of stay on ICU of < 1 week; five participants 1–2 weeks; three participants 2–3 weeks; eight participants 3–4 weeks; four participants 1–2 months; and one participant > 2 months (174 days).

**Table 1:** Distribution of participant ages (n = 25).

Age range	18–29 y	30–39 y	40–49 y	50–59 y	60–69 y	70–79 y
**Frequency**	1	3	4	4	8	5

The quantitative results from the ICU Memory Tool and EQ‐5D‐5L are reported in Table 2 and Table 3. All the participants reported some factual memories of their ICU stay on the ICU Memory Tool, with 22/25 also reporting delusional memories and 22/25 reporting some memories of feelings. These findings link to the significance of hallucinations, dreams, nightmares, and delusional and delirious experiences discussed by participants in the interviews.

**Table 2:** ICU Memory Tool results of participants after their treatment on ICU (n = 25).

	Frequency
Factual memories	25
Memories of feelings	22
Delusional memories	22
Thinking someone was trying to hurt or harm them	13
Any memories of ICU stay	25
Clear memories of ICU stay	3
Memories of:	
Family	18
Alarms	16
Voices	18
Lights	20
Faces	19
A breathing tube	14
Suctioning	12
Being uncomfortable	16
Darkness	13
A clock	12
A tube in the nose	17
Ward rounds	10
Feeling confused	16
Feeling down	11
Feeling anxious/frightened	19
Hallucinations	19
Nightmares	19
Dreams	15
Panic	15
Pain	13

**Table 3:** Baseline and post ICU discharge EuroQol 5 Dimension 5 Level (EQ‐5D‐5L) scores (n = 25). Values are median (IQR [range]).

	Before ICU admission	After ICU discharge
Mobility	1 (1–2 [1–4])	3 (1–3 [1–5])
Self‐care	1 (1–1 [1–3])	1 (1–2 [1–4])
Usual activities	1 (1–2 [1–3])	2 (2–3 [1–5])
Pain/distress	1 (1–2 [1–3])	2 (1–3 [1–4])
Anxiety/depression	1 (1–2 [1–3])	2 (2–3 [1–5])

On the EQ‐5D‐5L tool, 21/25 participants reported a decline in their health from before their ICU admission, with a decrease from a median (IQR [range]) visual analogue scale (VAS) score of 80 (60–90 [20–100]) before their ICU admission to 60 (35–75 [10–90]) (Table 3). Before ICU admissions the median EQ‐5D‐5L scores for mobility self‐care, usual activities, pain and anxiety/depression were all one. Post‐ICU the median mobility score was three, the median self‐care score was one and the median usual activities, pain, and anxiety/depression scores were all two. These findings reflect the transformative nature of being an ICU patient that was highlighted in the interview discussions, showing how different life can be for people after being patients on ICUs.

On the Good Death Measure, the items most identified as essential were that death be painless or largely pain free (16/25); that family and doctors follow the person's wishes (17/25); and that death be peaceful (15/25). In the interviews participants also commonly discussed the importance of death being pain‐free and peaceful, but also commonly indicated that dying at home and having loved ones present were also very important. The importance of family and doctors following the person's wishes was less commonly mentioned during participant interviews. Full Concept of a Good Death Measure results are in online Supporting Information Table S1.

Participants in this study, when asked to rate their experiences of ICU treatment using the Quality of Dying and Death tool as if it had been a dying experience, gave their experiences of ICU treatment a median (IQR [range]) Quality of Dying and Death score of 55/100 (38–74 [7–90]). Full questionnaire results are in online Supporting Information Table S2. The median (IQR [range]) chance of survival at which future ICU treatment would be acceptable to people who had previous been patients on ICUs was 30 (5–50 [1–90])%. One participant would not accept ICU treatment at any chance of survival.

The initial processes of data familiarisation and coding resulted in 341 codes identifying data points of potential analytic interest. These codes were initially clustered under five topic areas to generate initial themes: ‘Experiences of ICU treatment’; ‘Life after the ICU’; ‘End‐of‐life wishes’; ‘Dying in the ICU’; and ‘ICU decision‐making’. In light of further review of the full coded dataset, these were refined into three themes: ‘Safety, delirium and dependence: the positives and negatives of being a patient on an ICU, and the transformative effect of experiencing ICU treatment’; ‘The positive and negative possibilities involved in dying in the clinical context of an ICU’; and ‘The tension in shared decision‐making between the chance of survival and the goal of providing a good death’.

### Safety, delirium and dependence: the positives and negatives of being a patient on an ICU, and the transformative effect of experiencing ICU treatment

Participants described a range of positive and negative experiences associated with being a patient on an ICU. This was supported by the findings of the ICU Memory Tool. Positive experiences of ICU treatment mostly focussed on the excellence of ICU staff. The ICU was considered a place of safety, at one of the most dangerous times of their life:

*“That fantastically cocooned ICU where you know everything is being looked at, so you know, you're…terrifying to have been in there, I know, but very safe feeling that I knew I was looked after.”*

Participant 1

Interestingly, one participant who experienced multiple ICU admissions, described some as positive experiences and one as a negative experience:

*“I have had other [ICU] admissions which have been more positive, but I remember that one in particular feeling quite scared and a lot of the hallucinations and the things I was seeing were negative, quite nasty things.”*

Participant 11

Delirium, dreams, hallucinations and delusions were some of the most commonly remembered experiences of being a patient on an ICU. These involved altered sensory perceptions and fluctuating levels of consciousness. Some participants described how real events became incorporated into delirious experiences. Sedation was discussed as a contributing factor to these experiences and described as a very different experience to having a general anaesthetic:

*“I've obviously had anaesthetic many times before and again, it's not the same at all. And the assumption is that if you were going to go into an ICU, it would be similar, you go into hospital, you get sedated and that's what happens. No idea of any of the hallucinations.”*

Participant 46

Some participants reflected that people around patients on ICUs do not know what patients are actually experiencing:

*“I don't know how much can be done to control or not control but limit the risk of delirium, and I know there's a lot of research being done into that, but I don't think anybody could have known what was going on in my head.”*

Participant 48

Delirium experiences can feel so real that they were described as an alternate reality:

*“I would sometimes not be able to differentiate between reality and these dreams, they were so vivid. And at one point, I thought my husband had driven me to Canada and abandoned me. And it felt so real that I was mad at him for six months after I woke up, I felt like he abandoned me. And the memory of delirium is like a memory of an event, not a memory of a nightmare.”*

Participant 26

Several participants had experiences of thinking they were dying or dead or that people were trying to harm them:

*“I believed that a government organisation was trying to hunt down and kill my brother. And that they had captured me, and were torturing me to find out where he was. During that process, I believed that I was water‐boarded, that I had my wrists slit, and probes put in to shock me. I believed that my neck had been cut, for me to bleed out, in an attempt to get information, and that nanobots had been placed in my blood, with an attempt to control my actions. I believed I'd been taken all around the world, and, but the faces of the ICU staff were the various characters of this story of people who had been abusing me.”*

Participant 6

Being a patient in ICU was described as total dependence, being unable to speak or move, feeling powerless and trapped:

*“It's just being completely dependent on other people. It's a very… yeah, it's out of control, it's everything's been taken away from you, you're just so vulnerable and so scared and so weak and just so fragile.”*

Participant 44

*“Sometimes, you just feel like you're a thing on the bed, rather than a person.”*

Participant 6

Other negative experiences described included physical restraint, thirst, discomfort when a catheter was accidentally pulled out, difficulty breathing and fear:

*“It was absolutely terrifying, you know, and if I do have any terrifying moments, flashbacks or anything like that, it's that which I have. The feeling of not being in control and not being able to do the basics, such as breathing.”*

Participant 35

The experience of being an ICU patient was so negative for some participants that they wanted to die:

*“I remember the pain of that, and I remember begging to die. [My partner] said that I asked her to turn the machine off. I remember I asked a nurse to turn the machine off and I'm not religious, but I was praying: end it, end it, you know, because I couldn't stand the pain.”*

Participant 29

The experience of receiving ICU treatment was transformative [29] for many of the participants, involving changes to their perspectives, life goals and plans:

*“I think I am forever changed by this, and I think I can't stress that enough. But not all in a negative way. A lot of it, you could say, is negative, but it's not all negative in terms of I'm still here and talking to you about it. You know, there are positives as well that you never would've actually imagined could come out of a traumatic situation.”*

Participant 2

Participants experienced losing control over their own life and nearly dying whilst on ICU as a confrontation with mortality:

*“I think one of the takeaways that I've had from this is that you just don't know what's going to happen. I think before that, when I'd not really thought about death… and one of the things that I've learnt is life isn't like that, life is chaotic, unpredictable, traumatic. I just don't think you can plan in that way.”*

Participant 2

Participants described significant changes to their health and functional status following their admission to ICU, which was in agreement with the findings of the EQ‐5D‐5L questionnaire. They described processes of reflection, grieving and acceptance that were necessary for psychological recovery. Being a patient on ICU was also a confusing experience, which people needed to try and make sense of. There were several ways people attempted to do this, including reading their medical notes, ICU diaries, talking with family and looking at photos:

*“At one point I thought I was suspended on a big rope, a big metal…like a cable car cable. And it was coming into my arm, coming up my arm and out of my neck. And it was afterwards that I thought well, actually, I had a needle in my neck, didn't I, and I had a needle in my wrist, so I probably felt that this was some sort of cord that was connected. And that's… And the issues went away. Strangely, I was able to piece together those disjointed thoughts and sought comfort from them.”*

Participant 46

### The positive and negative possibilities involved in dying in the clinical context of an ICU

Participants were asked what they thought it might be like to die as an ICU patient, to elicit their reflections on ICU as a dying experience. Participants reflected that the experience of dying on ICU could potentially be similar to their previous experiences of being a patient on the ICU:

*“I think that actually dying is probably like what I went through. I think that's probably the beginning of it.”*

Participant 12

However, it was also acknowledged that the ICU dying experiences of different patients dying from different conditions are likely to be different:

*“I suppose again, it depends on what they were in for, how they were in, how long they were in, what's been done to them. … I mean, there could be, you know, sort of, a big drama or it could be, ‘pip’, and then it just goes silent.”*

Participant 35

Intensive care units were considered to have the potential to be good places to die, due to their highly trained staff, with appropriate privacy measures, pain control and sedation. It was also highlighted that dying on ICU allows for organ donation, which is important for the end‐of‐life wishes of some individuals:

*“I do also think that [ICU]s are environments where they're more used to dealing with people dying. And so actually maybe have really useful resources and people are able to do things like donate organs, which they otherwise wouldn't be able to, which they might take comfort from.”*

Participant 11

Intensive care unit gardens were also mentioned as ways that a good death can be promoted in ICU contexts:

*“So, they shut off the garden to everybody else, staff, and they allowed the family and that individual to go out, and they withdrew everything and let them die outside. And I just think that is lovely.”*

Participant 44

Conversely, dying on an ICU was also considered to be likely to be a traumatic way to die. Reasons given for this included the possibility of experiencing pain, including from invasive procedures:

*“It becomes burdensome to the dying person to have people poking, prodding, pushing, shoving things in you when you're on your way out, totally on your way out, or you're old. It looks violent, because it is violent, and the person in there, inside the dying person, experiencing the violence.”*

Participant 26

The possibility that dying on ICU could involve confusion, delusions and hallucinations was considered a terrifying prospect. The meant that dying on ICU had the potential to be out of keeping with participant's preferences that death be pain‐free and peaceful, as highlighted by the findings of the Good Death Measure:

*“I think to die after or during a sequence of that sort of dream would be horrendous. My father died on intensive care and if he had dreams anything like I had, I just hate to think how he'd have felt. No, I don't think anyone should have…if it's avoidable, I don't think anyone should have to go through that.”*

Participant 8

Participants reflected that delirium at the end‐of‐life could result in patients on ICUs looking peaceful whilst sedated but still experiencing hallucinations:

*“Let them have a quiet death because it might not be, inside their head, they may be screaming, we don't know.”*

Participant 35

Participants reflected that patients on ICUs might feel lonely at the end‐of‐life, as difficulty communicating could isolate a patient from their family:

*“If it was my last chance in the world to say anything, it would have been horrendous if I just couldn't get any message over anyway.”*

Participant 8

The lack of privacy and the clinical environment was felt to make dying with dignity difficult on ICUs:

*“It's a busy, high kind of stressful environment, and even with curtains around you do lack privacy. Even when in a side room, I think although better, you still lack privacy. So, I think if I wasn't aware of things, I don't think it would be a good experience. I wouldn't want that to be a lasting memory of me to my family and friends, kind of intubated, on a filter, you know, lines everywhere. I think that's not a nice way to go.”*

Participant 20

The possibility of dying on ICU being a negative experience was also informed by participant's remembering the bad deaths of other patients on the ICU:

*“The thing that I saw when I was in [ICU], when the patient ended up having a cardiac arrest and it was all very uncontrolled. And obviously the patient wasn't aware of any of that, but that just, it just looked so, and again, I don't know how much of that is reality, but it just looked so horrific. It didn't look, not that death necessarily looks nice, but having people just jumping on your chest and then just being a lot of people around and you're not, your loved ones aren't there. I think if that was the way, that would be quite, not a pleasant way of dying in an [ICU].”*

Participant 11

End‐of‐life care in the ICU was also considered likely to compare unfavourably to end‐of‐life care in hospices:

*“The staff I met in ICU I think would deal with it reasonably well. The difference between that and a hospice is that the hospice are specifically trained to deal with the death and dying bit.”*

Participant 16

### The tension in shared decision‐making between the chance of survival and the goal of providing a good death

Participants described a dichotomy between accepting ICU treatment and accepting death, characterised as a tension between the goals of survival and a good death. The involvement of advocates or palliative care input were considered necessary to protect patients from a system which is orientated to save lives and which could carry patients into unnecessary treatments against their wishes, overlooking their comfort at the end‐of‐life. Desiring survival was balanced against a reluctance to experience ICU treatment again:

*“The worst thing would be not having someone there to protect you from unnecessary procedures. … I feel like it's incompatible with the mission of intensive care people, put it that way. It's incompatible. You can't tell them to do everything they can to save somebody, and at the same telling them let them die. It's going to confuse them, and I don't know that they can make that fine distinction.”*

Participant 26

*“You couldn't give me any money in the world that would put me through that five or six days again.”*

Participant 38

The chance of survival was a key factor that participants felt impacted their ICU decision‐making, although many acknowledged that this can be difficult for doctors to predict:

*“As healthcare professionals you can't tell the patient if we give you this and that treatment there's a 95% chance you'll be absolutely fine again afterwards, because you can't say that, can you, or you can't say you'll be fine after six months or you'll be fine after five years, and that's what we want to hear as patients, and staff cannot give us that kind of answer.”*

Participant 48

Some participants described wanting to be assured that they had a good chance of survival before they would be willing to accept ICU treatment again:

*“There would have to be a pretty good chance of survival for me to want to be admitted to ICU.”*

Participant 20

Participants also discussed how the importance of survival differed depending on a person's stage of life. Some participants had a strong desire to pursue survival, due to having young dependants. Other participants without dependants and without outstanding life goals, felt that pursuing survival was not their top priority:

*“If it's one in one hundred, I've got to go through everything to see them boys, do you know what I mean. But you're asking a forty‐three‐year‐old, generally fit, firefighter. You ask a sixty‐five‐year‐old who has lived his life and he's happy, do you get me, I think that scale would go a bit higher.”*

Participant 38

Many participants felt that the decision to accept ICU treatment could not be made without knowledge of the clinical scenario. For example, the difference between accepting ICU treatment after elective surgery and accepting ICU treatment after an emergency critical illness was acknowledged:

*“What the big problem is, is people who've been in intensive care after heart surgery, or planned surgery, and they come bouncing in to me with a bunch of flowers and they go, oh, I was in for two days, and it was brilliant, and I was up and out, and I walked all the way down there, and I did this… And I didn't know for a long time that planned ICU is hugely different to your body.”*

Participant 1

Another factor that influenced decision‐making regarding accepting ICU treatment was the possibility of surviving with reduced functional status. The potential of functional decline had a range of effects on participants' willingness to accept ICU treatment, from having no impact on the decision, to survival with a reasonable quality‐of‐life being a vital consideration, especially maintaining cognitive capacity:

*“Were I to be given a diagnosis that would leave me with…I suppose the easiest way to sum it up is not as good quality of life as what I've got, then I wouldn't want treatment and would go down the palliative care route.”*

Participant 44

The importance of clarity and good communication from clinicians, especially about their chance of survival to guide their decision‐making, was discussed:

*“I think the one thing that's important, whether you've got the capacity to understand, is what…and NICE [National Institute of Health and Care Excellence] alludes to it, is shared decision‐making preferences and wishes.”*

Participant 36

Frustration was expressed about times information about prognosis or survival chance was not offered. Participants acknowledged that vulnerability and dependence due to critically illness could prevent patients from being able to participate in decisions about ICU treatment, resulting in a need to trust clinicians:

*“I probably wouldn't be able to make that decision anyway, would I, someone else would have to probably.”*

Participant 9

## Discussion

Study participants described positive and negative experiences of being an ICU patient. ICU staff were regarded highly by participants and many felt ICU was the safest place in the hospital. However, delirious memories and traumatic experiences of total dependence were also recalled. These experiences had transformative effects on the lives on participants and underpinned reflections that ICU treatment could cause negative dying experiences. This, along with the significant decline in health and function reported by participants, supports arguments for greater investment in both physical rehabilitation and psychological support services for patients after discharge from ICU [30].

The variability in experiences of ICU treatment led to variation in participants' reflections about dying on ICU and willingness to accept ICU treatment again. There were also differences in end‐of‐life wishes and willingness to talk about and plan for death. This variability suggests that study recruitment achieved inclusion of a good range of views within the dataset.

The questionnaire results indicate that participants experienced a significant reduction in their health‐related quality of life after their ICU admission, which is a common finding amongst ICU survivors [31]. The end‐of‐life wishes of participants were in keeping with the top scoring items in another study where the good death measure was used [25]. However, the participants in this study reported greater recall of their ICU treatment than in other studies [32, 33, 34], which could influence the findings of the study.

This study shows that people with experience of ICU treatment can express a chance of survival at which ICU treatment would not be acceptable to them. This finding is different to a previous study with people aged over 65 who received ICU treatment during COVID‐19, who were mostly unable to conceptualise what outcome thresholds would make future ICU treatment acceptable [35].

In an attempt to create a consensus statement about ICU triage, an international group of ICU clinicians were unable to agree on a survival cutoff for triage [36], suggesting that, in some situations, ICU treatment may be provided in the context of very low chances of survival and a very high chance of exposing the patient to a negative dying experience. Some authors have argued for the use of ICU treatments in context where there is no chance of survival, to allow families time to come to acceptance regarding the inevitable death of the patient [37]. The findings of this study challenge these approaches by highlighting that having an acceptable chance of survival is important to patients when considering whether to accept ICU treatment.

However, there were a wide range of ‘chances of survival’ at which ICU treatment was considered acceptable. The clinical implication of this is that ICU cannot be automatically considered to be acceptable to patients. Clinicians should ascertain the values of patients regarding the acceptability of ICU treatment, as there is no universally agreed position which can be assumed to hold, such as a desire to survive at all costs.

Several participants highlighted that people around patients on ICUs may be unaware what they are experiencing. Therefore, a novel use of the Quality of Dying and Death questionnaire was undertaken to try and assess what it is like to die on an ICU from a patient's perspective, which cannot be asked after patients have died. Participants of this study were asked to assess their ICU experience as if it was a dying experience using the Quality of Dying and Death questionnaire. Scores in this study were lower than the Quality of Dying and Death scores given by family members of people who had died in ICU in other studies [38, 39]. This could indicate a limitation with asking family or staff to assess patients' quality of death on ICUs, which may be better represented by ICU survivors' experiences. However, interpretation and comparison of these Quality of Dying and Death score findings is limited because the experiences assessed were ICU survivors' experiences, not dying experiences.

Strengths of the study include the qualitative methodology, which enabled a deep analysis of participant experiences of ICU treatment and their reflections on what it might be like to die on an ICU, as well as their willingness to accept ICU treatment. The mixed‐methods approach allowed for triangulation of the thematic analysis of the interview with the quantitative results of the questionnaire. The principal investigator's clinical background in anaesthesia (which involves exposure to ICU as a component of training) meant that the interviews were performed by someone with experience of ICU decision‐making, without the potential conflict of interest in being a practicing ICU clinician.

It is also important to note the limitations of the study. The sample size is relatively small, limiting the generalisability and transferability of the findings. However, participants were recruited from across the UK (and one from America), resulting in a breadth of locations and practices represented in the data. There is also a possibility of selection bias if participants with particular ICU experiences or perspectives were more likely to participate. There is also a risk of bias because the interviews were all done by a single interviewer, although the transcripts were read and coding discussed with the wider research team.

Whilst the nature of qualitative research means that results based on experiences of participants are not fully generalisable [19, 21], the study findings have significant theoretical implications that could inform future research and ICU decision‐making. They highlight a tension between the goals of survival and of providing a good dying experience in ICU decision‐making. Participants wanted shared decision‐making and for clinicians to provide information about the chance of survival. However, critical illness can prevent patients from participating in decision‐making, necessitating others making decisions for them, ideally informed by the patient's previous wishes and perspectives. Understanding the experiential burdens involved can inform the risk–benefit analysis of ICU treatment [40]. This study provides valuable information about the experiential harms of ICU treatment and the risk of a negative dying experience that should be weighed against the survival benefit that ICU treatment offers patients. Future research would be valuable to investigate whether the chance of survival that makes ICU treatment acceptable to people who have been patients on ICUs is affected by cultural/religious background or stage of life.

## Supporting information


**Table S1.** Concept of a Good Death Measure results.
**Table S2.** Quality of Death and Dying Questionnaire results.
