# Supplementary material for: Intensive care decision‐making, survival and dying well: a mixed methods study with people who have been intensive care patients
Source: Anaesthesia. 2026 Jul 9;81(10):1367–77. doi: 10.1111/anae.70285 (PMC13551127; doi:10.1111/anae.70285)
Supplement: Supplementary file 1 [file ANAE-81-1367-s001.docx]

**Table S1** Concept of a Good Death Measure results

| **Concept of Good Death Measure Item Frequencies** | **Essential** | **Important** | **Desirable** | **Not necessary** |
| --- | --- | --- | --- | --- |
| That death be painless or largely pain-free | 16/25 | 5/25 | 4/25 | 0/25 |
| That the dying period be short | 7/25 | 10/25 | 6/25 | 2/25 |
| That death be sudden and unexpected | 6/25 | 1/25 | 10/25 | 8/25 |
| That the family and doctors follow the person’s wishes | 17/25 | 6/25 | 2/25 | 0/25 |
| That death occur naturally, without technical equipment | 4/25 | 3/25 | 12/25 | 6/25 |
| That death be peaceful | 15/25 | 6/25 | 4/25 | 0/25 |
| That loved ones be present | 8/24 | 6/24 | 8/24 | 2/24 |
| The person’s spiritual needs be met | 4/25 | 5/25 | 10/25 | 6/25 |
| The person is able to accept death | 5/25 | 6/25 | 12/25 | 2/25 |
| A chance to complete important tasks | 6/25 | 10/25 | 6/25 | 3/25 |
| An opportunity to say “good-bye” | 7/25 | 8/25 | 9/25 | 1/25 |
| Able to remain at home | 2/25 | 4/25 | 12/25 | 7/25 |
| Lived until a key event | 1/25 | 3/25 | 10/25 | 11/25 |
| Death occurs during sleep | 2/24 | 7/24 | 11/24 | 4/24 |
| Mental alertness until the end | 4/25 | 6/25 | 10/25 | 5/25 |
| Control of bodily functions until death | 3/25 | 7/25 | 12/25 | 3/25 |
| Ability to communicate be present until death | 4/25 | 11/25 | 8/25 | 2/25 |

**Table S2** Quality of Death and Dying Questionnaire results (where 0 was a ‘terrible experience’ and 10 an ‘almost perfect experience’). Values are median (IQR [range])

| **Quality of Death and Dying Questionnaire Item** | **Value** |
| --- | --- |
| Pain under Control | 9 (8-10 [0-10]) |
| Control of Event | 3 (1-6.25 [0-10]) |
| Able to Feed oneself | 5 (1.75-6.75 [0-10]) |
| Control of bladder and bowels | 3 (1-9 [0-10]) |
| Able to breathe comfortably | 7 (5-9 [0-10]) |
| Energy to do things one wants to do | 2 (0-7.25 [0-10]) |
| Spend time with Children as much as you want | 6 (1-10 [0-10]) |
| Spend time with friends and other family as much as you want | 7 (3.75-10 [0-10]) |
| Spent time alone | 6.5 (1.75-9 [0-10]) |
| Be touched and hugged by loved ones | 8.5 (5.5-10 [0-10]) |
| Say goodbye to your loved ones | 8 (4.75-9.25 [0-10]) |
| Have the means to end your life if you need to | 0 (0-3.5 [0-10]) |
| Discuss your wishes for end of-life care with your doctor and others | 3.5 (0.75-8.25 [0-10]) |
| Feel at peace with dying | 6 (2-9 [0-10]) |
| Avoid worry about strain on your loved ones | 4 (2-9 [0-10]) |
| Be unafraid of dying | 8 (3-10 [0-10]) |
| Find meaning and purpose in your life | 5 (3-8.5 [0-10]) |
| Die with dignity and respect | 8 (6-10 [0-10]) |
| Laugh and smile | 8 (5.5-10 [0-10]) |
| Avoid being on dialysis or mechanical ventilation | 3.5 (1.75-7 [0-10]) |
| Location of death | 5 (1.5-8 [0-10]) |
| Die with/without loved ones present | 6 (4.5-8.25 [0-10]) |
| State at moment of death (awake/asleep) | 8 (5-10 [1-10]) |
| Have a visit from a religious/spiritual advisor | 7 (5-9 [0-10]) |
| Have a spiritual service or ceremony | 6 (3-8 [0-10]) |
| Have health care costs provided | 9 (6.25-10 [0-10]) |
| Have funeral arrangements in order | 7 (3.25-9.75 [0-10]) |
| Spend time with spouse, partner | 9 (7.25-10 [4-10]) |
| Spend time with pets | 1 (0-3 [0-10]) |
| Clear up bad feelings | 7 (3.5-8.75 [0-10]) |
| Attend important events | 2 (0-7 [0-10]) |
